# Prevalence of Metabolic Syndrome in Urban India

**DOI:** 10.1155/2011/920983

**Published:** 2011-05-19

**Authors:** Apurva Sawant, Ranjit Mankeshwar, Swarup Shah, Rani Raghavan, Gargi Dhongde, Himanshu Raje, Shoba D'souza, Aarti Subramanium, Pradnya Dhairyawan, Seema Todur, Tester F. Ashavaid

**Affiliations:** ^1^Research Laboratories, P. D. Hinduja National Hospital & Medical Research Centre, Mumbai 400016, India; ^2^Department of Preventive Medicine, Grant Medical College and Sir J. J. Hospital, Mumbai 400016, India; ^3^Department Laboratory Medicine, P. D. Hinduja National Hospital & Medical Research Centre, Veer Savarkar Marg, Mahim, Mumbai 400016, India

## Abstract

*Background*. Metabolic syndrome (MS) is characterised by a constellation of individual risk factors of cardiovascular disease. *Materials and Methods*. The current study was a population-based survey of cohort of subjects in the metropolitan city of Mumbai. A total of 548 subjects, who attended the CARDIAC evaluation camp, were recruited in the study. Participants with complete fasting lipid profiles, blood glucose, and known cardiac risk markers were evaluated. *Results*. On applying modified NCEP ATP III, we found out that nearly 95% of the subjects had at least one abnormal parameter. We found the prevalence of MS in our study population to be 19.52%. The prevalence of MS in males was almost double than females (*P* = .008). The overall prevalence of BMI (>23 kg/m^2^) was 79.01%. Increased hypertriglyceridemia and decreased levels of HDL-C were found to be more in males (*P* < .0001). *Conclusion*. The low percentage of subjects with normal and controlled parameters suggests that there is a need for awareness programs and lifestyle interventions for the prevention and control of MS.

## 1. Introduction

A global transition in the disease pattern has been observed, where the relative impact of infectious diseases is decreasing while chronic diseases like cardiovascular disease (CVD) and diabetes are increasingly dominating the disease pattern [1]. Epidemiologists in India and international agencies such as the world health organization (WHO) have been sounding an alarm on the rapidly rising burden of CVD for the past 15 years. It is estimated that by 2020, CVD will be the largest cause of disability and death in India, with 2.6 million Indians predicted to die due to CVD [2, 3]. 

The metabolic syndrome (MS) is a multiplex risk factor for atherosclerotic cardiovascular disease (ASCVD). It consists of an atherogenic dyslipidemia ((i.e., elevated triglycerides and apolipoprotein B (apo-B) and low high-density lipoprotein cholesterol (HDL-C)), elevation of blood pressure and glucose, prothrombotic and proinflammatory states. The risk of ASCVD accompanying the MS is approximately doubled compared with an absence of the syndrome. The MS appears to promote the development of ASCVD at multiple levels. Elevations of apoB containing lipoproteins initiate atherogenesis and drive lesion development. Atherosclerotic plaque development is accelerated by low levels of HDL-C, by elevated glucose levels and by inflammatory cytokines [4, 5].

MS is a complex web of metabolic factors that are associated with a 2-fold risk of CVD and a 5-fold risk of diabetes. Individuals with MS have a 30%–40% probability of developing diabetes and/or CVD within 20 years, depending on the number of components present [6].

In the United States (US), the prevalence of the MS in the adult population was estimated to be more than 25%. Similarly, the prevalence of MS in 7 European countries was approximately 23%. It was estimated that 20%–25% of South Asians have developed MS and many more may be prone to it [7, 8]. The main reason why MS is attracting scientific and commercial interest is that the factors defining the syndrome are all factors associated with increased morbidity and mortality in general and from CVD in particular [1].

In this context, the aim of this study was to assess the prevalence of MS as defined by NCEP ATP III guidelines with a modification to the value for BMI that is more applicable to the Asian Indian population, and to look for the differences between the various components constituting MS. Along with the prevalence of MS, we also studied the prevalence of various risk factors leading to ASCVD.

## 2. Materials and Methods

### 2.1. Study Design

The current study was a population-based survey of cohort in the metropolitan city of Mumbai in Western India. Mumbai being the industrial capital of India, the inhabitants here are heterogeneous both ethnically and culturally. A total of 560 subjects, who attended the free CARDIAC evaluation camp arranged by P. D. Hinduja National Hospital and Medical Research Centre by general advertising, were recruited in the study. Among the 560 subjects, 548 (302 males and 246 females) who had all the required data for the analysis formed the study group.

Each participant was interviewed by a group of research students and completed a standardized questionnaire containing information on demographics, anthropometric profile, individual characteristics associated with the major risk factors of CVD, past medical history, and biochemical parameters (Table 1). Prevalence of diabetes and hypertension was ascertained based on self-report of the physician's diagnosis and/or use of prescription medications along with medical records of therapeutics. All participants gave their written informed consent to participate in the study that was approved by the local ethics committee. 

### 2.2. Anthropometric Profile

All measurements were carried out by a group of research students. Body weight was determined with subjects wearing light clothes and no shoes or socks, using an electronic balance. Height was determined using a wall mounted, nonextendable measuring tape with subjects in standing position and feet together. Body mass index (BMI) was calculated using the expression:

(1) $$\text{BMI}=\frac{\text{weight}\;\left(\text{kg}\right)}{{\text{height}}^{2}\;\left(\text{m}\right)}.$$

### 2.3. Biochemical Analysis

Blood samples were collected by venipuncture after an overnight fast for 12–14 hours. Venous blood was collected in plain and fluoride bulbs for measurement of serum lipids, Lp(a), Apolipoproteins, hsCRP, and glucose, respectively. The serum was separated after centrifugation at 3000 rpm for 10 minutes. The analysis was carried on an automated clinical chemistry analyzer, Beckman DXC 800. Serum glucose was measured by oxygen rate method employing a Beckman oxygen electrode (glucose oxidase). Total cholesterol, triglyceride, and HDL-C concentration were measured by International Federation of clinical chemistry (IFCC) approved enzymatic methods. Beckman reagents and calibrators were used for the analysis. Control sera were included in each batch of samples analyzed. hsCRP detection is based on near infrared particle immunoassay on DXC 800. Serum APO A1, APO-B, and Lp(a) were determined by rate nephelometry on IMMAGE immunochemistry system. It measures the rate of increase in light scattered from particles suspended in solution as a result of complexes formed during an antigen-antibody reaction. As a part of external quality assurance, our laboratory is enrolled with the proficiency testing surveys of college of American pathologist (CAP) and is the 1st hospital laboratory in India to be CAP accredited.

### 2.4. Definitions and Preferred Cutoff Values

For serum lipids, we referred to National Cholesterol Education Program-Adult Treatment Panel III (NCEP-ATP III) guidelines [9]. According to these standard guidelines, hypercholesterolemia is defined as TC > 200 mg/dL, hypertriglyceridemia as TG > 150 mg/dL, and low HDL-C as <40 mg/dL. Dyslipidemia is defined by presence of one or more than one abnormal serum lipid concentration. For serum glucose levels, we referred to American Diabetes Association (ADA) guidelines [10]. Subjects with fasting blood glucose >100 md/dL were considered as having impaired blood glucose levels. For Lp(a) and hsCRP kit ranges were referred. For apolipoproteins A1 and B, we referred to IMMAGE instrument cutoff ranges. Obesity guidelines based on Western populations markedly underestimate the risk among all Asians because Asians have greater body fat at a given BMI [11] (Table 2). For BMI and abdominal obesity cutoff ranges, we referred to consensus guidelines for Asian Indians. Prevalence of metabolic syndrome in the cohort was assessed on the basis of the following criteria: the 2001 modified NCEP-ATP III-2 guidelines, wherein presence of any three of the following traits in the same individual would meet the criteria for MS:

1. abdominal obesity Men-≥90 cm, Women-≥80 cm,
2. serum TG > 1.69 mmol/L,
3. HDL-C < 1.03 mmol/L,
4. fasting blood glucose level >5.55 mmol/L,
5. blood pressure ≥ 130/85 mmHg.

### 2.5. Statistical Analysis

The statistical analysis was performed using Stata (SE 10.1 version). Results were expressed as mean ± standard deviation (SD). Pearson's chi-square test was applied to test the relationship of categorised independent and dependent variables. Odds ratio and their 95% confidence intervals were calculated for all 2 × 2 tables. A *P* value (significance) of <.05 is deemed statistically significant. A significance of  .000 should be read as *P* < .0001 (very highly significant) as the software can detect significance up to 3 decimal points only. Unpaired *T*-test (gender) and one-way analysis of variance (age) was performed. If ANOVA was significant, Bonferroni's *t* procedure was performed to assess difference between pairs. Prevalence of MS by means of its determinants was calculated using the prevalence rate formula: number of patients per total number of all subjects at the time of study multiplied by 100. Results were expressed as percentages.

## 3. Results

A total of five hundred and forty eight subjects participated in the study. On applying modified NCEP ATP III, consensus guidelines for defining obesity in Asian Indians and ADA, we found out that nearly 95% of the subjects had at least one abnormal parameter. The general characteristics of the study population are given in (Table 3).

### 3.1. Demographic Characteristics

The gender distribution was 56.75% males and 46.71% females. The age of the subjects ranged from 20 to 90 years, with a mean age in males of 54.28 years (SD = 13.89) and in females of 52.67 years (SD = 12.76). Of these, 18.65% males and 16.02% females were in 20–40 age group, 47.91% males and 57.42% females were in 41–60 age group, and 33.44% males and 26.56% females were >60 years old.

### 3.2. Anthropometric Profile

Our results showed a mean BMI of 25.68 in males and 26.95 in females with a 95% confidence interval (CI) of (25.27–26.09 in males) and (26.3–27.6 in females), which clearly shows that the prevalence of BMI ≥ 23 kg/m^2^ was significant in females than in males (*P* = .008). Both in males and females, the prevalence of overweight BMI (≥23 kg/m^2^) shows linear increase with age and was found to be more in males than females. The overall prevalence of BMI (≥23 kg/m^2^) was 79.01%. The prevalence of obesity was high in 41–60 age group females than 20–40 age group and >60 age group. The prevalence of obesity is almost the same in 20–40 and 41–60 age group males but drops down as age advances. The incidence of abdominal obesity observed was 70.9% and waist to hip ratio was 73.76%.

### 3.3. Lifestyle or Behavioural Determinants

Our results showed that around 15% of the participants in the study were alcoholic and smokers. The prevalence of smokers and alcoholic was highly significant in males, as compared to females. In males 41–60 age group showed high prevalence of smokers and alcoholic as compared to 20–40 and >60 age groups.

### 3.4. Clinical Analysis

It was found in the current study that history of hypertension and diabetes increases as age advances both in males and females. Prevalence of history of diabetes was significant in males than in females (*P* = .015). Prevalence of history of hypertension in both males and females was highly significant in 41–60 age groups (*P* < .0001). History of diabetes in 41–60 age group males was highly significant (*P* < .0001). Prevalence of family history of cardiovascular diseases was observed in 27.76% subjects.

### 3.5. Biochemical Analysis

#### 3.5.1. Lipids

Increased fasting blood glucose, hypertriglyceridemia and decreased levels of HDL-C were found to be more in males with high TG and low HDL-C to be highly significant (*P* < .0001). Hypercholesterolemia was highly significant in females as compared to males. Both males and females in 41–60 age groups showed significantly high levels of impaired glucose levels (*P* < .0001). On further comparing agewise, prevalence of low HDL-C in 20–40 age group males was 64.91% which is very high as compared to other age groups both in males and females. In males, the prevalence of hypercholesterolemia, and hypertriglyceridemia was found to be more in 41–60 age group. In females, fasting blood glucose, hypertriglyceridemia and hypercholesterolemia showed a linear increase with age.

#### 3.5.2. Cardiac Markers

Percentage prevalence of APO B and APO B/APO A1 ratio was found to be more in males than females. Prevalence of APO A1 was approximately similar in males and females. High levels Lp(a) and hsCRP was seen in females than in males. In females, Lp(a) and hsCRP prevalence was seen more in young adults (20–40 age group) than the other two groups under study.

### 3.6. Metabolic Syndrome

**Figure 1:**
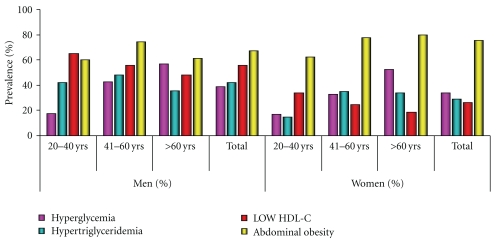
Age- and gender-specific prevalence of individual components of MS.

The overall prevalence of MS having ≥3 components was 19.52% by modified NCEP ATP III criteria. The prevalence of MS in males was almost double (25.16%) than females (12.6%), and this was highly significant (*P* = .008). The agewise distribution of prevalence of MS was found to be the same in 20–40 and 41–60 age groups (20.61% and 20.76%), respectively, whereas >60 age group showed a marginal decrease in the prevalence (16.66%). The prevalence of individual components of MS is reported in (Figure 1). 

### 3.7. Prevalence of Risk Factors of ASCVD

**Figure 2:**
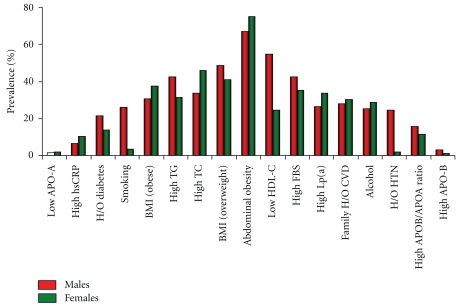
Gender-specific prevalence of different atherosclerotic risk factors.

The prevalence of major risk factors of ASCVD was 45.25% overweight, 33.75% obese, 39.96% having impaired blood glucose levels, 39.96% subjects with hypercholesterolemia, 38.13% with hypertriglyceridemia, and 47.97% with low HDL-C. The prevalence of elevated cardiac markers was 2.18% with high APO B, 1.82% with increased APO A, 30.65% and 8.39% with elevated levels of Lp(a) and hsCRP, respectively (Figure 2). 

## 4. Disscussion

Asian Indians are a high risk population with respect to diabetes and CVD, and the numbers are consistently on the rise [12]. The prevalence of MS in Asian Indians varies according to the region, the extent of urbanization, lifestyle patterns, and socioeconomic/cultural factors. Recent data show that about one third of the urban population in India's major cities have MS [13]. The NCEP definition is more flexible as it can diagnose MS even in the absence of glucose intolerance, which in itself is a predisposition to dysmetabolic dyslipidemia, an obesity phenotype and proinflammatory status. The prevalence of MS by modified NCEP ATP III criteria in the current study showed gender-specific differences. Our results illustrate marked heterogeneity in the prevalence of MS according to gender. The prevalence of MS in our study in males was 2 times higher as compared to females, whereas in other studies in India, MS prevalence in women was 1.5–2 times higher than in males [14, 15]. A higher prevalence in men might be related to their higher rates of overweight BMI, impaired blood glucose levels, high TG, and low levels of HDL-C.

Chow et al. [16] found a prevalence of MS of 26.9% in males and 18.4% in females in southern India which is in concordance with our results. We found the prevalence of MS in our study population to be 19.52%, which corroborate with Deepa et al. [17] CURES 34 study showing prevalence of 18.3%. The prevalence of MS did not change with respect to age difference, 20–40 and 41–60 age groups showed similar prevalence of MS, and a marginal decrease was seen in >60 age group. 

The development of obesity, or more specifically an increase in abdominal fat, is thought to be the primary event in the progression of MS. A tendency to gain fat in the abdominal area, as opposed to the hip, buttock, and limb areas, is linked to a rise in fatty acids in the blood, which is thought to lead to insulin resistance, high blood pressure, abdominal blood lipids, and eventually diabetes. Asian Indians tend to develop central obesity rather than generalised obesity. About three fourth of the subjects participated in study were overweight/obese (BMI ≥ 23 kg/m^2^), being a prime determinant of MS prevalence. Of these around one third of overweight/obese subjects had impaired glucose tolerance and many exhibit features of MS.

In our study, we observed approximately 15% of subjects had history of diabetes. This means that the remaining subjects with impaired blood glucose levels that is, 25% of subjects were on their way to develop diabetes, which is an important risk factor for CAD. Enas et al. [18] in Coronary Artery Disease in Indians (CADI) study report the prevalence of diabetes to be 3–6 times higher among South Asians than Europeans, Americans, and other Asians. In India, it is estimated that 32 million people suffer from diabetes, and the number is projected to increase to 69.8 million by 2025 [12].

Increased prevalence of low HDL-C has been reported earlier by Enas et al. [18] who found that only 4% of Asian Indian men and 5% Asian Indian women had optimal HDL-C levels. Low HDL-C levels are a strong predictor of occurrence and reoccurrence of Myocardial infract (MI) and stroke and are associated with premature and severe CAD. Approximately half of the population had low levels of HDL-C of which 65% were males from 20–40 age group that is, the young adults. Similar findings were also reported by Sawant et al. [19] a recent study on 9000 subjects (<40 years of age) attending the Health Check program at P. D. Hinduja National Hospital, it was shown that around 64.2% men and 33.8% women had abnormally low levels of HDL-C. Obesity reduces HDL-C levels, and obese patients with MS and atherogenic dyslipidemia almost always have low HDL-C levels. Our study shows that around 35% of subjects had low HDL-C were either overweight or obese.

The novel aspect of this study is that along with clinical and biochemical features of MS, prevalence of risk factors for ASCVD with cardiac markers was systematically assessed. Along with traditional risk factors, emerging factors present in the South Asian population are allowing for further CAD risk stratification. At the forefront is APOB/APO A1 ratio. APOB is the major apolipoprotein found in low density lipoprotein (LDL), Intermediate-density lipoprotein (IDL), and very low density lipoprotein (VLDL), and it is the primary ligand for the LDL receptor. APO A1 is the major protein constituent of HDL. The APO B/APO A1 ratio provides an atherogenic to antiatherogenic lipoprotein ratio that has been shown to be a better predictor of CVD than LDL level and HDL level. The APO B/APO A1 ratio can identify individuals with preponderance of small dense LDL particles [8]. In our study, 15.76% males and 11.33% females were found to have abnormal APO B/APO A1 ratio.

Lipoprotein a (Lp(a)), considered an emerging risk factor by NCEP ATP III, has been implicated in the development of the premature atherosclerotic disease seen in South Asians [8]. Among patients with Lp(a) excess, the CAD risk is increased by 3-fold in the absence of other risk factors. The risk increases to eightfold with low HDL-C, 12-fold with high LDL-C, 16-fold with diabetes and 25-fold with TC/HDL-C ratio. The higher the Lp(a) level the lower the age of first heart attack, and the most affected individuals develop MI by the third to fifth decade of life. High levels of Lp(a) correlate with the prematurity, severity, extent, and progression of coronary atherosclerosis as well as the occurrence and recurrence of MI among Asian Indians [12]. In our study, 26.37% of males and 33.73% females showed elevated levels of Lp(a).

The cardiovascular field has recently shown great interest in the role of inflammation in the development of ASCVD. The basic concept is that atherogenesis represents a state of chronic inflammation. The findings that elevations of serum CRP carry predictive power for the development of major cardiovascular events led to the concept that advanced and unstable atherosclerotic plaques are in an even higher state of inflammation than stable plaques. In our study, females (10.2%) had higher rates of elevated CRP than males (6.43%). It is of interest that obese persons and particularly those with metabolic syndrome also have elevated levels of CRP [20]. It was found that of the total subjects with elevated CRP 86.95% were found to be obese.

The prevalence of MS in the present study is much lower than that reported in an earlier study in urban Indian adults aged (20–50 years) in which the prevalence was reported to be 41.1% [21]. The prevalence of MS based on ATP III criteria in Jaipur (urban north Indian population) was 24.9%. In Chennai urban population Study (CUPS) [22], the prevalence of MS as defined by EGIR was found to be 11.2%. In the study of 10 industrial settings, prevalence of MS was 26.6% [3]. Among Asian Indian, Immigrants living in US showed the prevalence of MS to be 32%. These findings suggest that there is a significant difference even within an urban environment in different socioeconomic groups. 

The high levels of risk observed with our baseline cross-sectional survey justify the need for establishing a surveillance system to monitor the trends of MS and ASCVD risk factors over time. Continuing surveillance efforts would provide us with an opportunity to develop evidence-based cost-effective CVD prevention, detection, and management strategies. Early intervention particularly with lifestyle change would delay the onset of advance forms of MS and a high-risk status. Regular physical activity, lifetime abstinence of tobacco, avoidance in the consumption of trans fats (fried food), and reduced intake of saturated fats together with an increased intake of fruits and vegetables form the foundation of lifestyle changes. Adopting a healthy lifestyle beginning in childhood and adolescence is warranted in view of the malignant nature of CAD among Asian Indians [12, 23]. 

## 5. Conclusion

Clinical diabetes and CAD are preceded by a constellation of risk factors that are also the components of MS. In conclusion, prevalence of MS varies amongst ethnic groups. Indians are high at risk for CVD and their predispositions. The prevalence of MS was double in males as compared to females. This study revealed the increased prevalence of MS to be more prevalent in 41–60 years, suggesting that this group is at increased risk of developing CAD. It was also found that high percentage prevalence of overweight and obesity was one of the major driving forces in the development of MS. Therefore, early identification of the metabolic abnormalities and appropriate intervention may be of primary importance in populations especially like ours having high prevalence.

## 6. Limitation

Due to missing/unavailability of blood pressure data, we were not able to include it in the present study, but would try to incorporate it later when we obtain complete information on the same. As cluster of three or more, risk factors can be termed as metabolic syndrome, in the current study as per NCEP ATP III guidelines abdominal obesity, HDL-C levels, triglyceride levels, and fasting blood glucose levels were taken into consideration.

## Figures and Tables

**Table 1:** Study variables for the baseline survey of CVD risk factors.

Study variables	
Demographic	Age
	Gender
Behavioural	Smoking
	Alcohol consumption
Clinical examination	Weight
	Height
Questionnaire	History of CVD, diabetes, HTN
	Family history of CVD, diabetes, HTN

Biochemical tests	Fasting blood glucose
	Total cholesterol (TC)
	Triglyceride (TG)
	HDL-C
	APOA1, APOB. RATIO
	Lp(a)
	hsCRP

(HTN-hypertension, APO A1-apolipoprotein A1, Lp(a)-lipoprotein A, hsCRP-High sensitive C-reactive protein).

**Table:** (a) Lipid profile

	Normal (mmol/L)	High (mmol/L)
Cholesterol (TC)	<5.18	≥5.18
Triglycerides (TG)	<1.69	≥1.69

	Normal (mmol/L)	Low (mmol/L)

HDL-C	≥1.03	<1.03

**Table:** (b) Cardiac markers ranges

	Normal	High
Lp(a)	≤1.07 *μ*mol/L	>1.07 *μ*mol/L
hsCRP	≤0.75 mg/dL	>0.75 mg/dL
APOB-MALES	<1.62 g/L	≥1.62 g/L
APOB-FEMALES	<1.71 g/L	≥1.71 g/L

	Normal	Low

APOA-MALES	≥0.90 g/L	<0.90 g/L
APOA-FEMALES	≥1.07 g/L	<1.07 g/L
APOA/APOB ratio	≤1	>1

**Table:** (c) Glucose levels

	Normal (mmol/L)	High ((mmol/L)
FBS	<5.55	≥5.55

**Table:** (d) Body mass index

	Normal (kg/m^2^)	Overweight (kg/m^2^)	OBESE (kg/m^2^)
BMI	18.5–22.9	23–24.9	≧25

**Table 3:** The general characteristics of the study population.

Characteristics of the study population				
Characteristics	Males *n* (%)	Females *n* (%)	Total	*P* value
*Age (years)*				
20–40 YRS	58 (18.65)	41 (16.02)	99 (17.46)	
41–60 YRS	149 (47.91)	147 (57.42)	296 (52.20)	.075
>60 YRS	104 (33.44)	68 (26.56)	172 (30.34)	

*BMI*				
Normal	62 (20.53)	53 (21.54)	115 (20.99)	
Overweight	147 (48.68)	101 (41.06)	248 (45.26)	.169
OBESE	93 (30.79)	92 (37.4)	185 (33.76)	

*Smoking*				
Non-smokers	195 (74.14)	243 (96.43)	438 (85.05)	<.001*
Smokers	68 (25.86)	9 (3.57)	77 (14.95)	

*Alcohol*				
No alcohol consumption	235 (75.56)	251 (98.05)	486 (85.71)	<.001*
Alcohol consumption	76 (24.44)	5 (1.95)	81 (14.29)	

*HTN*				
Normal	233 (71.70)	179 (69.92)	402 (70.90)	.642
H/O HTN	88 (28.3.)	77 (30.08)	165 (29.10)	

*Diabetes*				
Normal	244 (78.46)	221 (86.33)	465 (82.01)	.015**
H/O diabetes	67 (21.54)	35 (13.67)	102 (17.99)	

(*-significant).
